# Effect of nanoshell geometries, sizes, and quantum emitter parameters on the sensitivity of plasmon-exciton hybrid nanoshells for sensing application

**DOI:** 10.1038/s41598-023-38475-1

**Published:** 2023-07-13

**Authors:** A. Firoozi, Angela Amphawan, R. Khordad, A. Mohammadi, T. Jalali, C. O. Edet, N. Ali

**Affiliations:** 1 Department of Physics, College of Sciences, Yasouj University, Yasouj, 75918 Iran; 2 Smart Photonics Research Laboratory, Sunway University, 47500 Sunway, Selangor Malaysia; 3 Future Cities Research Institute, Sunway University, 47500 Sunway, Selangor Malaysia; 4 Department of Physics, Persian Gulf University, Bushehr, 75196 Iran; 5 Institute of Engineering Mathematics, Universiti Malaysia Perlis, 02600 Arau, Perlis Malaysia; 6 Faculty of Electronic Engineering Technology, Universiti Malaysia Perlis, 02600 Arau, Perlis Malaysia; 7 Department of Physics, Cross River University of Technology, Calabar, Nigeria; 8 Advanced Communication Engineering (ACE) Centre of Excellence, Universiti Malaysia Perlis, 01000 Kangar, Perlis Malaysia

**Keywords:** Nanoscience and technology, Optics and photonics

## Abstract

A proposed nanosensor based on hybrid nanoshells consisting of a core of metal nanoparticles and a coating of molecules is simulated by plasmon-exciton coupling in semi classical approach. We study the interaction of electromagnetic radiation with multilevel atoms in a way that takes into account both the spatial and the temporal dependence of the local fields. Our approach has a wide range of applications, from the description of pulse propagation in two-level media to the elaborate simulation of optoelectronic devices, including sensors. We have numerically solved the corresponding system of coupled Maxwell-Liouville equations using finite difference time domain (FDTD) method for different geometries. Plasmon-exciton hybrid nanoshells with different geometries are designed and simulated, which shows more sensitive to environment refractive index (RI) than nanosensor based on localized surface plasmon. The effects of nanoshell geometries, sizes, and quantum emitter parameters on the sensitivity of nanosensors to changes in the RI of the environment were investigated. It was found that the cone-like nanoshell with a silver core and quantum emitter shell had the highest sensitivity. The tapered shape of the cone like nanoshell leads to a higher density of plasmonic excitations at the tapered end of the nanoshell. Under specific conditions, two sharp, deep LSPR peaks were evident in the scattering data. These distinguishing features are valuable as signatures in nanosensors requiring fast, noninvasive response.

## Introduction

Bio-nanosensors reveal a patient's physiological health status through the noninvasive detection of clinically relevant biomarkers in biofluids without complex manipulation^1^. In healthcare, the discovery of distinguishing properties of biomarkers in new materials play a significant role in the development of rapid noninvasive bio-nanosensors for medical diagnosis and monitoring of patients^2,3^. During a pandemic, rapid detection of infectious diseases is important to effectively prevent the spread of the disease to other patients^4^. Minopoli et al.^5^, discussed the various types of nanostructures used in plasmonic biosensors including nanoparticles, nanoholes, nanorods, and nanoshells for various applications, such as disease diagnosis, drug discovery, and environmental monitoring. Nguyen et al.^6^, present a novel approach to tune the plasmonic coupling of gold nanorods using the M13 bacteriophage as a biomaterial actuator, with potential applications in sensing, imaging, and energy conversion. Aldewachi et al.^7^, describe the fabrication and functionalization of AuNPs as colorimetric biosensors, emphasizing their potential for sensitive and rapid detection of analytes in complex samples and integration with other technologies for various bioanalytical applications including the detection of proteins, nucleic acids, small molecules, and pathogens. Kim et al., present a novel approach to the co-assembly of thermo-plasmonic nanoparticles and colloidal quantum dots into three-dimensional plasmonic nanoclusters with enhanced photothermal and photoluminescence properties. This approach could have potential applications in various fields, such as photothermal therapy, bioimaging, and energy conversion^8^.

Surface plasmon polaritons (SPPs) are electromagnetic waves that propagate along the interface between a dielectric and a metal, evanescently confined in the perpendicular direction. Localized surface plasmon resonance (LSPR) is stimulated by an incident free-space optical signal when it encounters nanoparticles smaller than its own wavelength. The LSPR can create strong electromagnetic fields, known as hot spots, which can be exploited for various applications such as sensing and imaging. The excitation of LSPR depends on the various parameters including size, material, shape, and RI of surrounding media of nanostructures. These parameters play an important role in determining the peak wavelength of the LSPR. By choosing the proper values of these key parameters, the LSPR peak can be tuned to the desired wavelength range of interest.

The LSPR of metallic nanoparticle depend on the material properties, such as the dielectric constant, which determines the strength of the interaction between the incident light and free electrons on plasmonic nanoparticle. In this paper we used silver due to its high extinction coefficients and low absorption at visible and near-infrared wavelengths and due to these properties, Ag nanoparticle has various application including, biosensing, enhancing solar cell efficiency and so on.

The RI of the surrounding medium affects the LSPR by altering the interaction between the incident light and the plasmonic nanoparticle. The resonant wavelength of the LSPR shifts to longer wavelengths with increasing RI of the surrounding medium. This effect can be used for sensing applications.

The shape of plasmonic nanoparticles determine the distribution of the electromagnetic field and the location of the hot spots. Different shapes of plasmonic nanoparticles such as spheres, nanorods, cone, truncated cone and rounded cone can create different hot spots and field enhancements, leading to different LSPR properties. For example, cone like plasmonic nanoparticles have stronger and more localized hot spots than spherical nanoparticles due to their anisotropic shape.

The size of the nanoparticle affects the LSPR properties by influencing the number of free electrons on the nanoparticle surface and their collective oscillations. Small nanoparticles have fewer free electrons and weaker LSPR signals than larger nanoparticles. However, smaller nanoparticles have higher sensitivity to changes in the surrounding environment, making them suitable for sensing applications.

In recent years, many studies have been conducted on nanomaterials stimulating the localized surface plasmon (LSP) effect to investigate biomarkers of various diseases such as Covid-19, SARS, tuberculosis, cancer, HIV, Alzheimer's, sclerosis and more^9–14^. LSP biosensors have a small footprint, making them suitable for wearables and portable medical diagnostic devices for patient monitoring^15–19^.

The investigation of the interaction between LSP in plasmonic nanoparticles (PNs) and excitons in two- and three-level quantum emitters (QEs)^20–22^ has received much attention in both theoretical and experimental investigations^23–28^. It is known that the LSP around plasmonic nanoparticles leads to a very strong electromagnetic field around the nanoparticles. This strong field is a few nanometers away from the nanoparticles. Thus, optical properties of QEs in the vicinity of plasmonic nanostructures can be modeled and investigated^29–34^. The enhancement in spontaneous emission upon coupling between PNs and QEs is called weak coupling. Induced transparency also occurs in the middle region, and Rabi splitting occurs in the strong region^35–39^. The plasmon-exciton interaction, the induced transparency and also the strong coupling can be well studied with classical, semi-classical and quantum models^40–54^.

The change in the refractive index (RI) of tissues in patients is often used as an indicator of the severity of a disease^14,16–20^. This was done using fiber Bragg grating sensors, prism coupling refraction and imaging approaches^14,16–20^. The approaches rely on variations in spectral properties due to scattering or absorption and do not respond well to low concentrations of patient biomolecules. Similarly, LSP-based sensors using spherical nanoparticles do not respond well to ambient RI changes at low concentrations of biomolecules due to the broad width of the plasmon resonance peak. Significant research has been conducted on the design and fabrication of nanosensors that are highly sensitive to changes in the environment, including pseudomaterials, silver-core and gold-coated nanoshells, multilayered nanoparticles, etc.^55–57^. To address the limitations of spherical LSP nanoparticles, we combine exciton in QEs with plasmonic nanoparticles to form plasmon-exciton nanostructures that are more sensitive to RI changes in the patients. In this work, nanosensors based on plasmon exciton (plexciton) coupling shall be designed and modeled, the effects of all parameters affecting the optical properties of nanosensors shall be investigated, as well as tuning the surface plasmon resonance peak to the desired range.

The optical response exhibits uniquely strong coupling signature once the coupling strength between QEs exceeds all quenching rates in a hybrid system at zero detuning conditions, namely the Rabi splitting of an assumed resonant mode that forms two new hybrid states, namely upper and lower polaritons. Conventional systems involving the optical response of QEs typically assume these emitters to be simple interacting systems^50,58^ or coupled Lorentz oscillators^59^. For example, such models have recently been used to predict and adopt an innovative phenomenon of transparency induced by a strong dipole–dipole interaction^60–67^. The Study of the interaction between molecular excitations and metallic surface plasmon resonances (SPPs) has improved significantly, both theoretically and computationally. However, the consequences of this mutual feedback are not well studied, especially in systems containing both metallic nanostructures and semiconductor or molecular particles or layers.

One of the proposed ways to increase the accuracy and efficiency of LSP-based sensors are plexciton nanostructures in QEs, which are a very good candidate for this sensor type^68–70^. The types of structures used in this earlier work include nuclear cladding, arrays of plasmonic structures or holes, photonic nanoantennas in conjunction with an absorber. To extend the previous work on plasmonic exciton nanostructures, in this paper, we design and model a plasmon-exciton hybrid nanoshell that comprises a core of metal nanoparticles and a coating of molecules. The structure is based on the coupling of LSP in metal nanoparticles and excitons in the molecule and controls the optical responses in the desired range. In this work, we design and model a nanosensor based on plexciton coupling to analyze geometric effects on optical properties of the nanosensors and the surface plasmon resonance peak. In order to achieve this, the Maxwell-Bloch equations are first solved on the basis of the FDTD method^71–73^. Subsequently, the optical properties of plasmonic nanoparticles are analyzed for different nanostructures. The influence of different parameters on the optical response of these structures is investigated to identify distinguishing features for nanosensors.

## Solution of Maxwell-Bloch equations using FDTD

The FDTD method is mainly used in classical electrodynamics. This method can be employed to solve the Maxwell-Bloch (MB) equations to investigate the coupling of quantum systems (including molecules or atoms) with plasmonic nanostructures. The issue of coupling quantum systems with plasmonic nanostructures can be studied in a semi-classical way. For this purpose, the interaction of light with plasmonic nanostructures is solved by Maxwell equations and quantum systems with Liouville equations. The MB equations are simulated by discretizing space and time. The following equations are used

1 $$\frac{{\partial {\varvec{E}}}}{\partial t} = \frac{1}{{\varepsilon_{0} }}\vec{\nabla } \times {\varvec{H}} - \frac{1}{{\varepsilon_{0} }}\frac{{\partial {\varvec{P}}\left( {r,t} \right)}}{\partial t}$$

2 $$\frac{{\partial {\varvec{H}}}}{\partial t} = - \frac{1}{{\mu_{0} }}\vec{\nabla } \times \user2{E }$$

where ${\varvec{P}}\left(r,t\right)$ is the polarization in position $r$ and time $t$. The polarization is given by

3 $${\varvec{P}}\left( {r,t} \right) = n_{0} \left\langle {\varvec{\mu}} \right\rangle = {\text{Tr}}\left[ {\rho \left( {r,t} \right){\varvec{\mu}}} \right]$$

Here, $\rho \left( {r,t} \right)$ is the density matrix of the system. The density matrix is used to study the properties of quantum systems. The density operator is expressed by

4 $$\hat{\rho } = \mathop \sum \limits_{i = 0}^{k} p_{i} \left| {\left. {\psi_{i} } \right\rangle } \right.\left. {\left\langle {\psi_{i} } \right.} \right|$$

where ${p}_{i}$ is the probability of the state $\left| {\left. {\psi_{i} } \right\rangle } \right.$. The time evolution of the density operator is given by

5 $$i\hbar \frac{{\partial \hat{\rho }}}{\partial t} = \left[ {\hat{H},\hat{\rho }} \right] - ik\Gamma \hat{\rho }$$

where $\hat{H}$ and $\Gamma$ are the total Hamiltonian and the relaxation operator, respectively. In this work, quantum systems with two-level emitters are investigated. We present the density matrix elements related to two-level systems that we simulate semi-classically with FDTD.

In studying the interaction of light with quantum systems, two-level systems are less complex than three-level and higher systems. Therefore, it is easier to study the optical properties and understand the optical phenomena. The Hamiltonian of this system has perturbation and non-perturbation parts due to the interaction of the incident light with the quantum system. The non-perturbation Hamiltonian and the interaction potential of the two-level system are given by

6 $$H_{0} = \mathop \sum \limits_{j = 0}^{N - 1} \hbar \omega_{j} \left| {\left. j \right\rangle } \right.\left. {\left\langle j \right.} \right|$$

7 $$V_{int} \left( {\vec{r},t} \right) = \mathop \sum \limits_{j = 0}^{N - 1} \hbar \Omega_{j} \left( {\vec{r},t} \right)\left[ {\left| {\left. 0 \right\rangle } \right.\left. {\left\langle j \right.} \right| + \left| {\left. j \right\rangle } \right.\left. {\left\langle 0 \right.} \right|} \right]$$

where $\Omega_{j} \left( {\vec{r},t} \right)$ is the Rabi frequency is related to the interaction between the field and the emitter. It is expressed by

8 $$\Omega_{j} \left( {\vec{r},t} \right) = \frac{{\mu_{0j} \vec{E}\left( {\vec{r},t} \right)}}{\hbar }$$

By using above equations, the elements of the density matrix are given by

9 $$\dot{\rho }_{00} = \mathop \sum \limits_{j \ge 1} i\Omega_{j} \left( {\vec{r},t} \right)\left( {\rho_{0j} - \rho_{j0} } \right) + \Gamma \rho_{jj}$$

10 $$\dot{\rho }_{jj} = i\Omega_{j} \left( {\vec{r},t} \right)\left( {\rho_{0j} - \rho_{j0} } \right) + \Gamma \rho_{JJ}$$

11 $$\dot{\rho }_{0j} = i\Omega_{j} \left( {\vec{r},t} \right)\left( {\rho_{00} - \rho_{jj} } \right) + \left( {i\omega_{0j} - \gamma } \right)\rho_{0j}$$

Therefore, the FDTD method with MB equations is a powerful tool that can be used to simulate the interaction of light with QEs. By combining the FDTD method with the MB equations, it is possible to simulate the interaction of light with QEs in the presence of a biosensor. This can be used to analyze the performance of biosensors, which are devices that use light to detect the presence of specific molecules.

### Model configuration and simulation layout

The FDTD method is a numerical technique that can be used to solve Maxwell’s equations in time and space. The MB equations are a set of coupled differential equations that describe the interaction of light with matter. By combining the FDTD method with the MB equations, it is possible to simulate the interaction of light with QEs, such as atoms and molecules. This can be used to analyze the performance of biosensors, which are devices that use light to detect the presence of specific molecules.

The simulation layout for the FDTD method with Maxwell-Bloch equations for QEs to analyze a biosensor as a test case is as follows: the computational domain is divided into two regions: the bulk region and the sensor region. The bulk region is used to model the propagation of light in the absence of the biosensor. The sensor region is used to model the interaction of light with the biosensor. The QE is placed in the sensor region. The simulation is run for a number of wavelengths to obtain the absorption spectrum of the biosensor.

**Figure 1:**
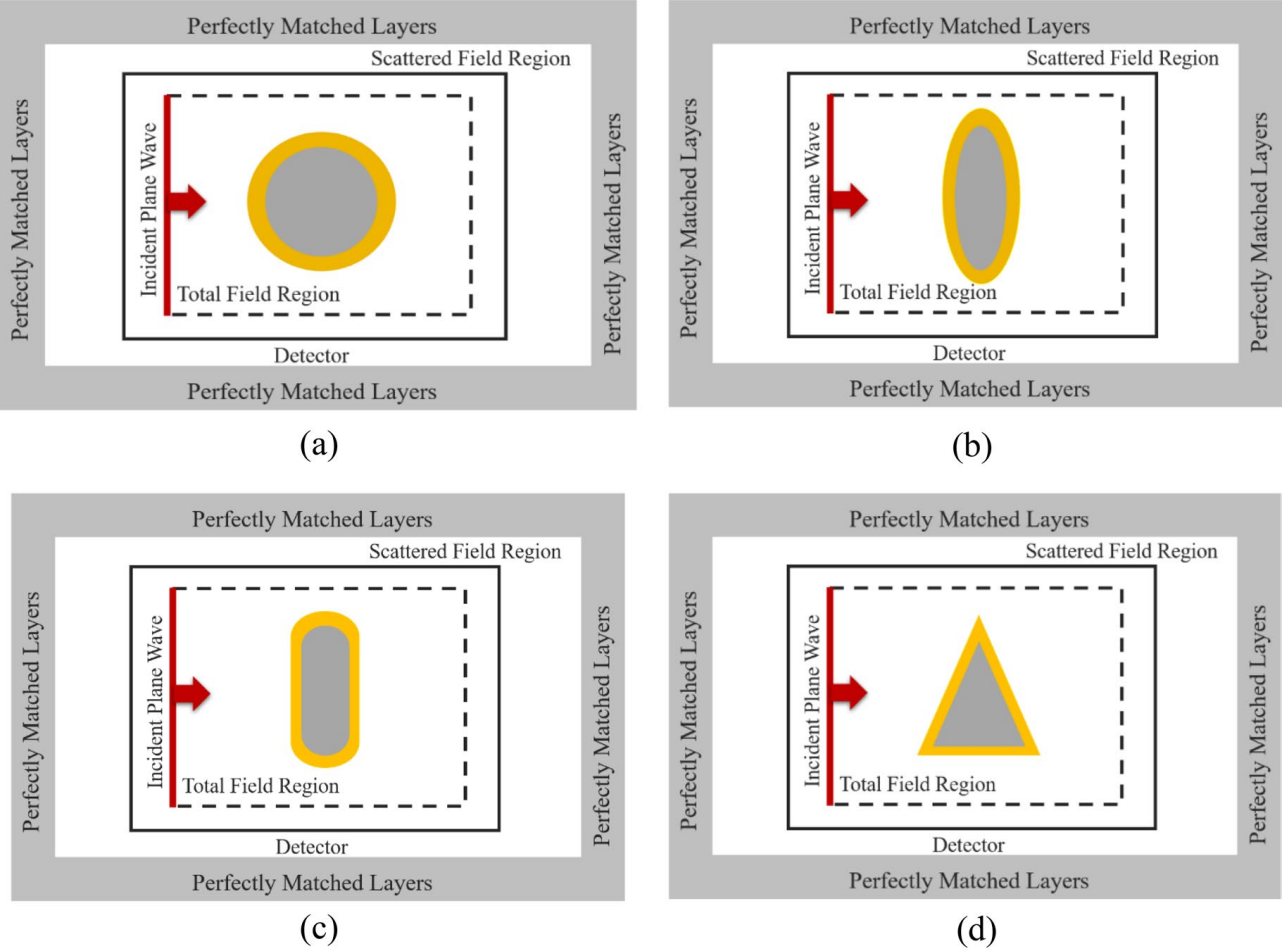
Simulation space for calculating the scattering cross section of (**a**) spherical nanoshell, (**b**) elliptical nanoshell, (**c**) rod nanoshell, and (**d**) cone like nanoshell.

The model configuration for the FDTD method with Maxwell-Bloch equations for QEs to analyze a biosensor as a test case is as follows: QE parameters are, the atomic dipole moment 10D, the number density $8 \times 10^{24} \;\;{\text{m}}^{ - 3}$, total coherence rate 1 THz. A TE polarized plane wave (the magnetic field normal to the simulation space) is injected using the total field-scattered field technique (TF/SF)^71^. The reason for choosing the wave with this polarization is the ability to excite the LSPR in plasmonic nanoparticles. To get accurate results in the 3D FDTD method, fine meshes are required. In this paper we calculate our result with mesh size = 1 nm. The computational domain is divided into a mesh of cells. The Maxwell equations are solved in each cell to determine the electromagnetic field. The MB equations are solved in each cell to determine the state of the QE. The interaction between the electromagnetic field and the QE is calculated. The simulation is repeated for a number of time steps to obtain the time evolution of the electromagnetic field and the QE. Nanoshells, composed of a core of metalic nanoparticles and a coating of molecules based on LSP coupling, are among the nanostructures that optical responses in the desired region. The designed nanoshells with different structures in the simulation space are shown in Fig. 1a–d. In all cases we considered nanoshell composed of Ag core and a shell with a thickness of 5 nm of the QE, embedded in water with RI of 1.33.

## Results and discussion

The FDTD method is a powerful tool that can be used to simulate the interaction of light with matter. By combining the FDTD method with the MB equations, it is possible to simulate the interaction of light with QEs, such as atoms and molecules. The simulation can be used to determine the emission spectrum of the quantum dot, as well as the efficiency of the emission process. This can be used to analyze the performance of nanosensors, which are devices that use light to detect the presence of specific molecules. To design and fabricate nanosensors based on plexciton coupling, the effects of all parameters influencing the optical properties of nanosensors as well as tuning the surface plasmon resonance peak to the desired range should be studied. Therefore, in the following, the optical properties of plasmonic nanoparticles are first examined and then various nanostructures are modeled. The influence of different parameters on the optical response of these structures is also investigated to design an optimal nanosensor.

**Figure 2:**
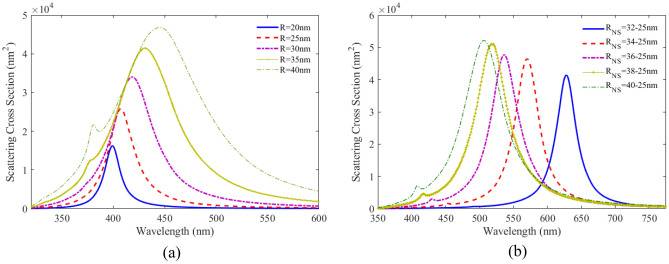
SCS as a function of wavelength for (**a**) spherical Ag nanoparticles for different radii, (**b**) spherical nanoshell composed of dielectric core with a RI of 1.46 and Ag shell for different core radii, embedded in water with RI of 1.33.

To investigate the optical properties of plasmonic nanoparticle and studied the effect of geometrical parameter, we considered spherical nanoparticle made of silver in water with the RI of 1.33. The 3D FDTD simulation space for calculating the optical properties is the same as Fig. 1a. We use Drude Lorentz dispersion model to simulate the optical constant of gold, which was fitted to optical data from Johnson and Christy. Figure 2 shows the scattering spectra versus wavelength for Ag nanoparticles with different radii. As can be seen, the peak of localized surface plasmon resonance (LSPR) shifts toward longer wavelengths, and the width of the curves enhances with the increasing radius of Ag nanoparticle. This increase in width in the optical response of nanoparticles prevents the nanoparticles from performing well as nanobiosensors.

Then, we considered spherical nanoshells consisting of dielectric core of silica with a RI of 1.46 and silver shell in water with a RI of 1.33. The SCS of the structure is calculated for different shell radii and is shown in Fig. 1b. It is observed from the obtained result that the peak position of LSPR shifts to lower wavelengths as the shell thickness increases. Comparing the scattering of the nanoshell with the Ag nanoparticles, it is found that the peak shifts of the LSP in the designed nanoshell covers a broader range of wavelengths, and the width of the peak is less than the silver spherical nanoparticles. Comparing the optical properties of plasmonic nanoparticles with plasmonic nanoshells, it is concluded that the structure of nanoshells is suitable for applying plasmonic nanosensor.

### Light matter interaction in plexciton spherical nanoshells

**Figure 3:**
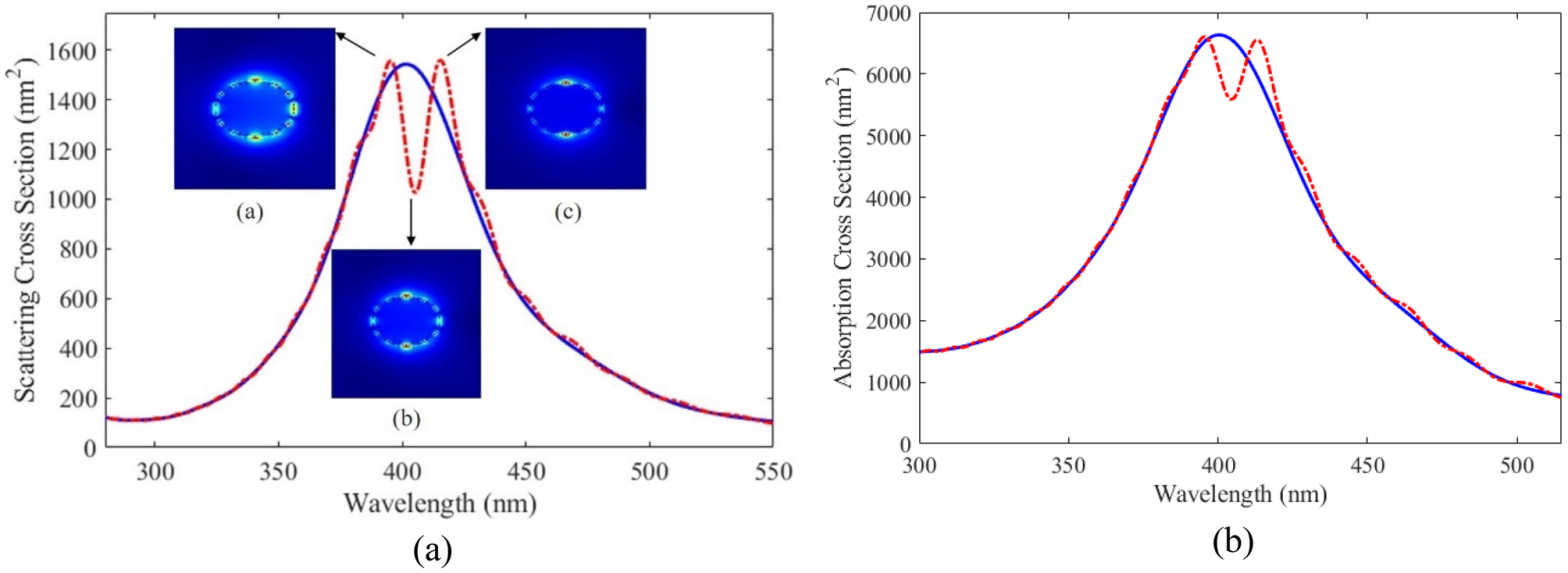
(**a**) SCS and (**b**) absorption versus wavelength for spherical Ag nanoparticles (solid line) and plexciton spherical nanoshells (dashed line) consisting of Ag core with a radius of 20 nm coated with a 5 nm of QE. The inset of (**a**) shows the electric field profiles at wavelength of (**a**) 395 nm, (**b**) 407 nm and (**c**) 414 nm.

Based on the results of the previous section, we propose plexciton hybrid nanoshells with various geometry for sensing application. First of all, we consider plexciton spherical nanoshell consisting of Ag core with a radius of 20 nm coated with a 5 nm of QE. The wavelength of QE is 407 nm. The simulation space for calculating the optical properties of plexciton hybrid spherical nanoshell is shown in Fig. 1a. The effect of key parameters is examined on the nanostructures. Figure 3a shows the scattering spectrum of plexciton hybrid nanoshell as a function of wavelength (dashed line). To better understand the effect of QE on optical properties of nanostructure, the scattering spectra of Ag nanoparticle versus wavelength is also plotted in Fig. 3a (solid line). The plasmon-exciton hybrid nanoshell exhibits notably different scattering spectrum compared to scattering cross section of Ag nanoparticle. It is seen that the maximum scattering spectrum of Ag nanoparticles occurs at 407 nm. The scattering spectrum of the nanoshell shows a plasmonic splitting and dip due to the coupling between QE with plasmonic nanoparticles. The optical response of plexciton nanostructures, which is a hybrid system of plasmonic and excitonic components, are influenced by various factors including plasmon-exciton coupling, plasmon and exciton resonances, environmental factors, and size and shape variations.

The interaction between the plasmonic and exciton can lead to the formation of new hybrid modes, known as plexcitons. The energy and intensity of these modes depend on the strength of the plasmon-exciton coupling, which can be tuned by adjusting the distance between the plasmonic and excitonic components. Understanding these factors is essential for designing and optimizing plexciton nanostructures for various applications, such as sensing, imaging, and energy conversion.

To investigate the underlying physics of plexciton coupling in the proposed nanostructure, the absorption cross-section of plexciton hybrid nanoshell and also Ag nanoparticle are calculated and the results are shown in Fig. 3b. As can be seen from the obtained results, plasmonic splitting and a dip is clearly occurred at the same wavelength as the scattering spectrum. These plasmonic splitting and dip in absorption cross-section spectrum confirm the coupling between QE with plasmonic nanoparticles. The two split peaks and dip are located at 395 nm, 414 nm, and 407 nm, respectively. The optical field profiles at these wavelengths are shown in the inset of Fig. 3a. These electric field distributions around the proposed nanostructure demonstrate that the plasmonic splitting is caused by the coupling between the quantum emitter shell and Ag core.

**Figure 4:**
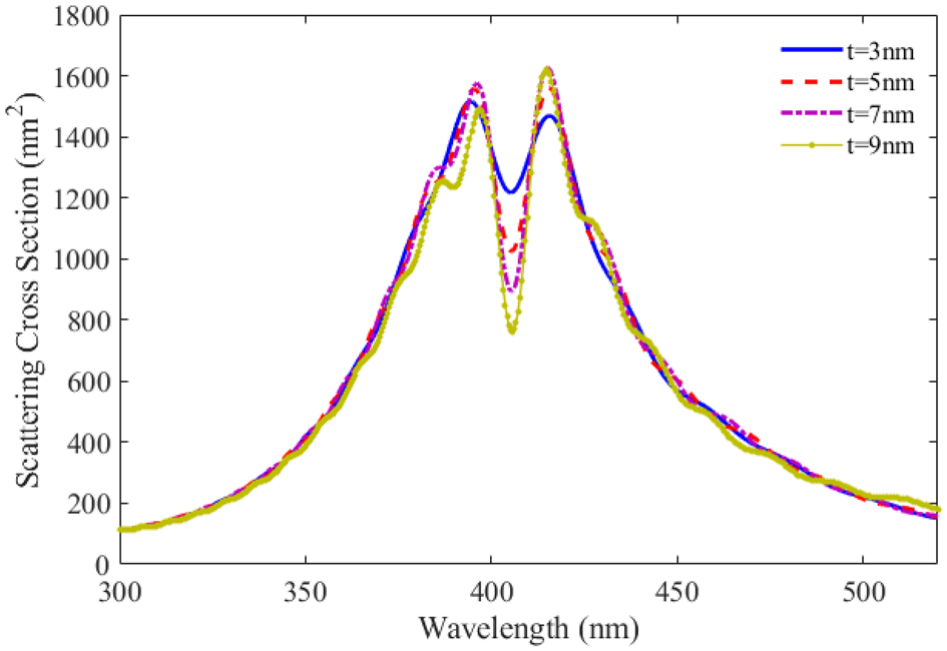
SCS versus wavelength for plexciton spherical nanoshell with different shell thicknesses.

In order to investigate the effect of the sizes of the proposed plexciton nanoshell on its optical response, the scattering spectra for different shell thicknesses (t) is calculated. The results are shown in Fig. 4. It can be observed that observed that the dip of the scattering reduces as the thickness of the QE increases.

**Figure 5:**
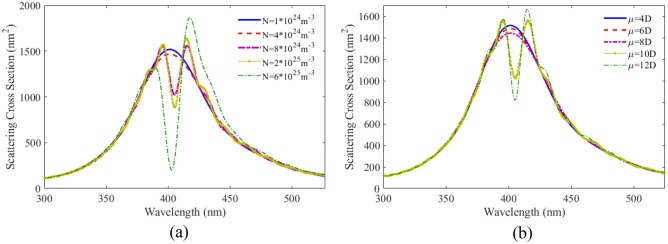
SCS versus wavelength for plexciton spherical nanoshell with different (**a**) number densities (**b**) dipole moments of QE.

It is to be noted that the QE parameters also have significant influences on the optical response of the proposed nanostructure. For this purpose, the effect of number density is investigated on the scattering spectra and the results is shown in Fig. 5a. It can be seen that with increasing number density, the minimum depth at the curves increases and shifts to lower wavelengths.

Another important parameter is the dipole moment of the QE. The scattering spectra of plexciton hybrid nanoshell for various dipole moments has been plotted in Fig. 5b. As can be seen for dipole moments less than 10D, we do not observe any plasmonic splitting and a dip. However, above this value, a dip is observed in the curves. With increasing the atomic dipole moment, the dip becomes deeper. Spherical nanoshells are the most common type of nanoshell. They are relatively easy to synthesize and have a wide range of applications. However, they have relatively low sensitivity.

### Plasmon − Exciton Interactions in elliptical nanoshell

**Figure 6:**
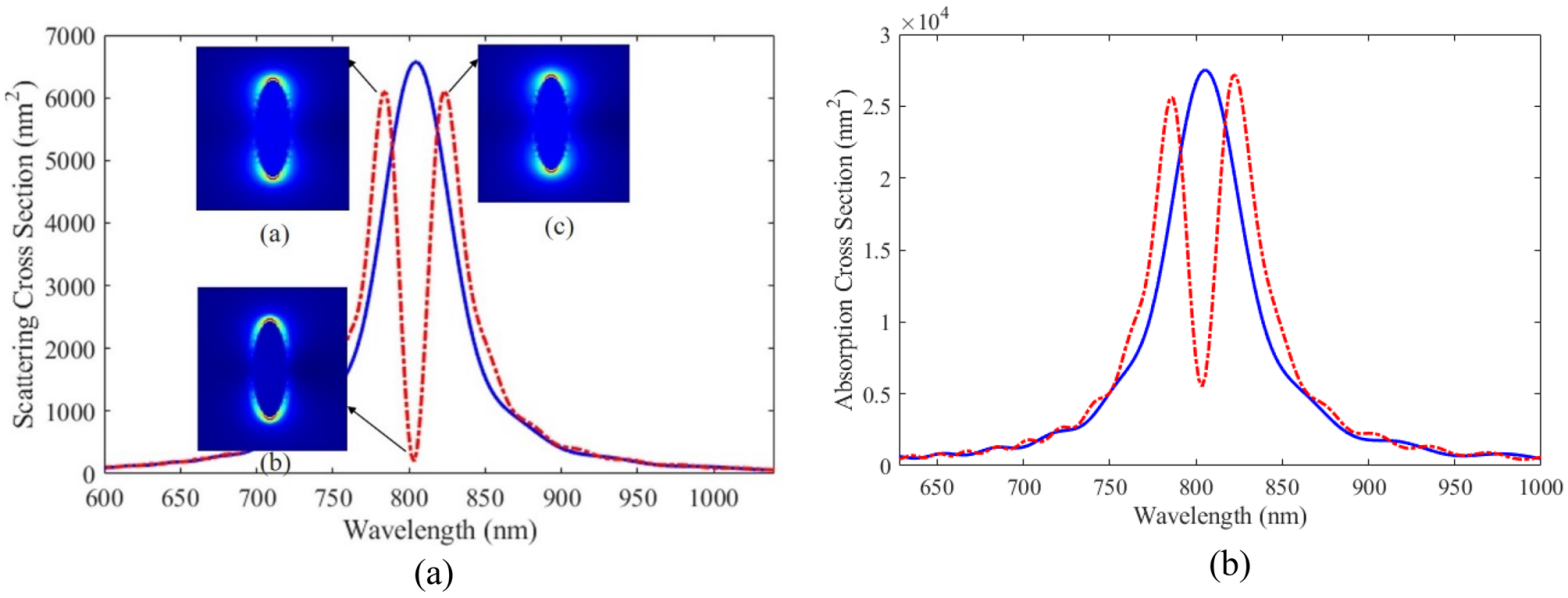
(**a**) SCS and (**b**) absorption versus wavelength for elliptical Ag nanoparticles (solid line) and plexciton elliptical nanoshells (dashed line) consists of Ag core with a small radius of 10 nm and a large radius of 40 nm coated with a 5 nm of QE. The inset of (**a**) shows the electric field profiles at wavelength of (**a**) 783 nm, (**b**) 802 nm and (**c**) 823 nm.

The advantage of the proposed nanostructure compared to the previous one for sensing application is that it has more key parameters to adjust the resonance peak in the wavelength range of interest, such as geometrical parameters of the core and shell and also QE parameters (including number density and different dipole moments). To accurately set the resonance peak to the desired range and increase the interaction of plasmonic nanoparticles and QEs, the proper choice of the mentioned parameters is of great importance. The plexciton hybrid nanoshell with elliptical geometry consists of Ag core with a small radius of 10 nm and a large radius of 40 nm coated with a 5 nm of QE. The wavelength of QE is 804 nm. Figure 6a shows the scattering spectrum of Ag elliptical nanoparticle (solid line) and the proposed plasmon-exciton elliptical nanoshell (dashed line) as a function of wavelength. It can be seen that compared to the scattering spectrum of plexciton spherical nanoshell, scattering spectrum of elliptical nanoshell has a deeper dip. It shows the coupling between QE with plasmonic nanoparticles. The scattering spectrum exhibits two split peaks and a dip, occurring at 783 nm, 802 nm, and 823 nm, respectively. The inset of Fig. 6a displays the electric field profiles around the plexciton elliptical nanoshell at these wavelengths, revealing an electromagnetic interaction between the quantum emitter shell and Ag core. By comparing the electric field profiles around the plexciton elliptical nanoshell and a spherical nanoshell, we find that the plasmon-exciton coupling is stronger in the case of the plexciton elliptical nanoshell.

In order to examine the result more precisely, the absorption cross section of Ag elliptical nanoparticle (solid line) and the proposed plexciton elliptical nanoshell (dashed line) as a function of wavelength is plotted.in Fig. 6b. As expected, the behavior that occurred at the scattering spectra is also observed at the absorption cross section. It is clear that the dip occurs at a particular wavelength and has small width. It also shows that another key parameter that can play an important role to adjust surface plasmon resonance peak in the desired range is the geometry of nanoshell. We continue to study the effect of important parameters such as RI, core radius, shell thickness, and QE parameters including emitter dipole moment and number density, on the optical response of this nanostructure.

**Figure 7:**
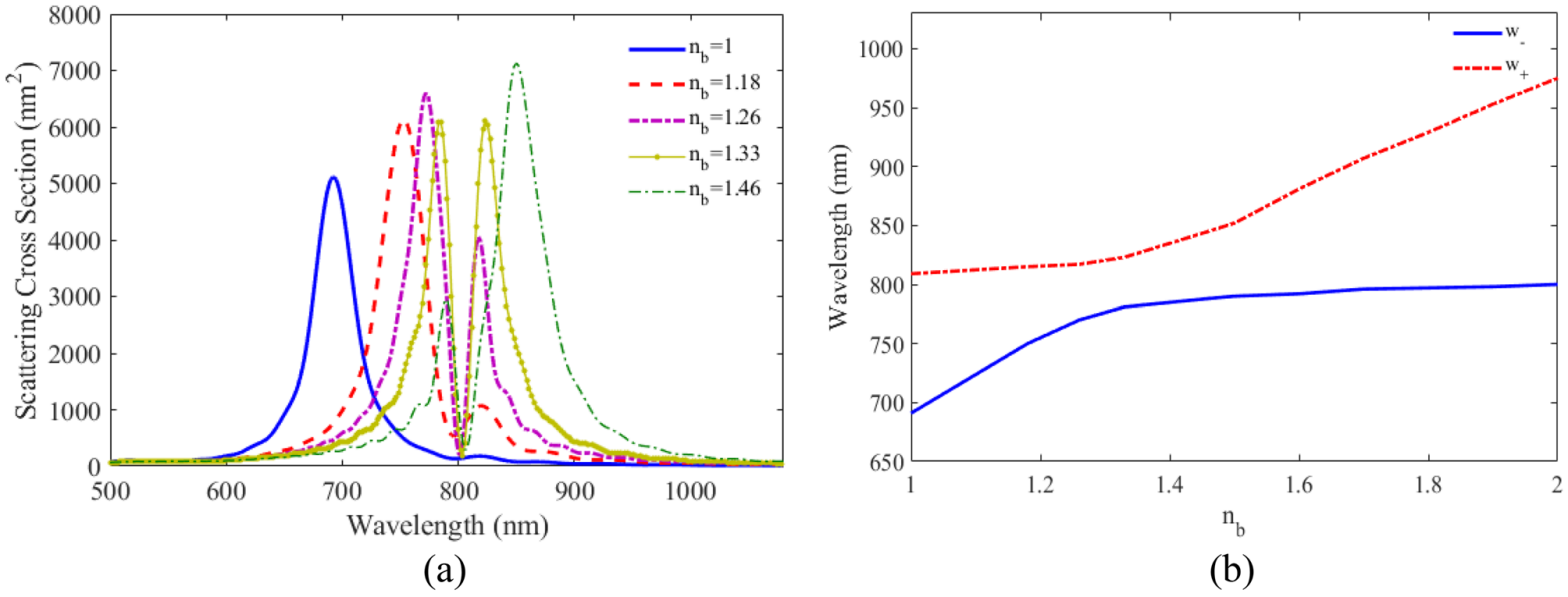
(**a**) SCS versus wavelength for plexciton elliptical nanoshell with different RI of the environment. (**b**) The variation of maximum wavelengths versus RI of plexciton elliptical nanoshells.

To investigate the performance of the proposed plexciton elliptical nanoshell as nanosensor, the optical response of these nanostructure with various RIs of surrounding media (n_b_) is calculated and the calculated result is shown in Fig. 7a. It can be observed that with the increase of n_b_, the intensities of the two peaks change with opposite trends and two peaks position shift to longer wavelengths. For more detailed study, Fig. 7b illustrates the variation of the wavelengths of the two split peaks scattering cross-section of plexciton elliptical nanoshell versus the RI of the surrounding media from 1 to 2. It is seen that the wavelength of two peaks redshift without crossing the 802 nm, which is the absorption wavelength of QE.

**Figure 8:**
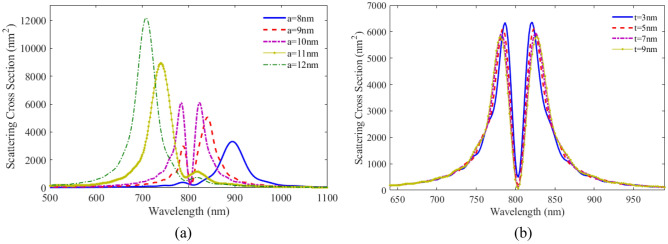
SCS as a function of wavelength for plexciton elliptical nanoshells with different (**a**) small radii of core, (**b**) shell thicknesses.

To investigate the effect of geometrical parameters on optical response of the proposed plexciton nanoshell, first we study the effect of small radius of core and then the shell thickness. In Fig. 8a, the scattering spectra is plotted as a function of wavelength with different small core radius. It can be seen that as the small core diameter decreases, the peak position of LSPR shifts to longer wavelengths and plasmon-exciton coupling decreases. Figure 8b shows the SCS versus wavelength for the plexciton nanoshell with different shell thicknesses. As the shell thickness increases, the dip in the figure becomes deeper, and the scattering height reduces at longer thickness.

**Figure 9:**
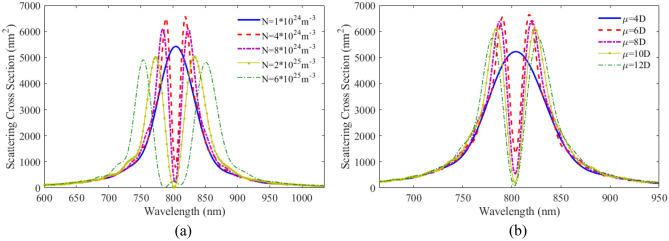
SCS versus wavelength for plexciton elliptical nanoshell with different (**a**) number densities, (**b**) dipole moments of QE.

The effect of number density and dipole moment are also studied. In Fig. 9a the scattering spectra for the plexciton elliptical nanoshell is plotted with different number densities. As can be seen, the coupling between PNs and QEs is not strong at a density below $$4 \times 10^{24} \;\;{\text{m}}^{ - 3}$$$$.$$ As the number density increases, the coupling increases and thereby the distance between the two peaks becomes farther apart. The height of the scattering spectra also decreases and the width of the two peaks increases. Comparing the scattering results of plexciton elliptical nanoshell with a plexciton spherical nanoshell, it is found that the coupling between QE and the plasmonic nanoparticles is higher in the former case. Figure 9b shows the SCS versus wavelength for the plexciton elliptical nanoshell with different dipole moments. As can be seen, the coupling between the emitter and the nanoparticles is in the weak region for the 4D dipole moment. The coupling increases with increasing dipole moment. Elliptical nanoshells have a higher sensitivity than spherical nanoshells. This is because the elliptical shape allows for more efficient coupling of light into the plasmonic mode.

### Plasmon-exciton coupling in rod nanoshells

To study the effect of different geometry on optical response of plexciton nanoshell, we consider rod nanoshell. The Ag core parameters are: a height of 80 nm and a radius of 10 nm coated with a thickness of 5 nm of the QE. The wavelength of QE is 792 nm. The proposed plexciton nanostructure together with the simulation space is shown in Fig. 1c.

**Figure 10:**
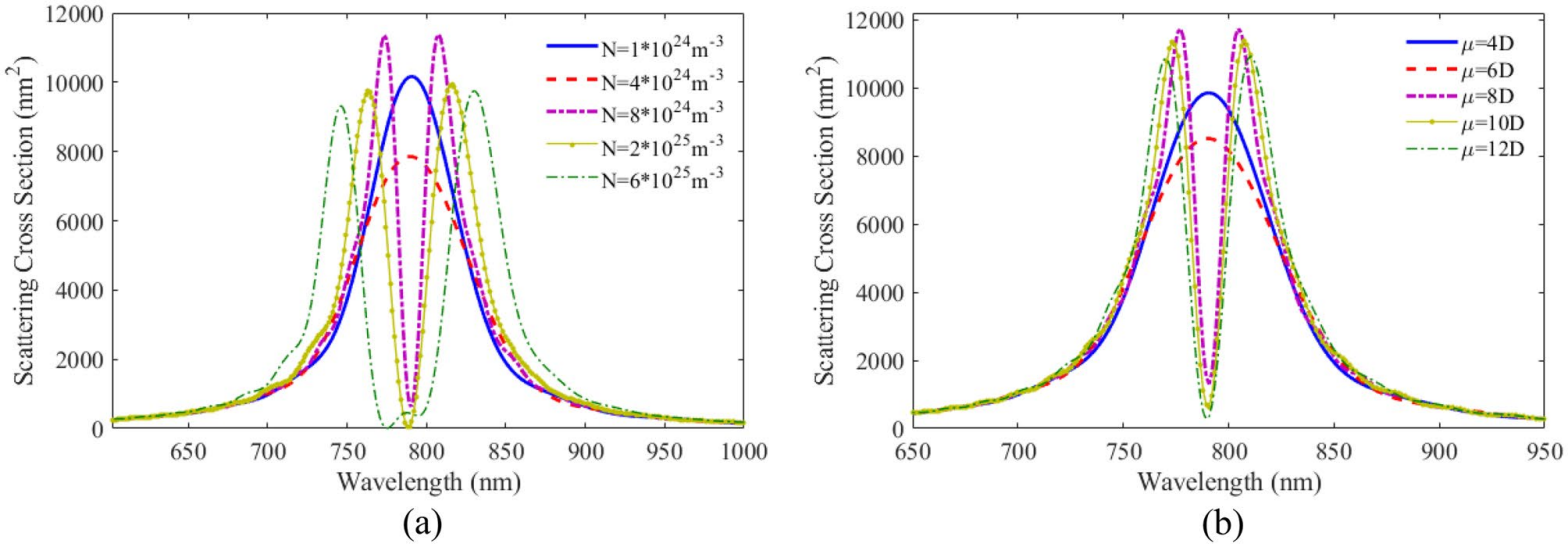
SCS versus wavelength for plexciton rod nanoshell consists of Ag core with a height of 80 nm and a radius of 10 nm coated with a 5 nm of QE with different (**a**) number densities, (**b**) dipole moments of QE.

Figure 10a shows the scattering spectra versus wavelength for plexciton rod nanoshell with different number densities of QE. The calculated result show that, like a spherical nanoshell, at a number density of less than $8 \times 10^{24} \;{\text{m}}^{ - 3}$, the coupling between the nanoparticle and the QE is weak. Figure 10b shows the scattering spectra as a function of wavelength for rod nanoshells with different dipole moments of QE. Comparing the results from rod-shaped nanoshells and spherical nanoshells, it can be seen that the behavior of these two geometries is similar for atomic dipole moments smaller than 8D. But in the case of a higher dipole moment, the effect of this geometry on the coupling is more significant.

**Figure 11:**
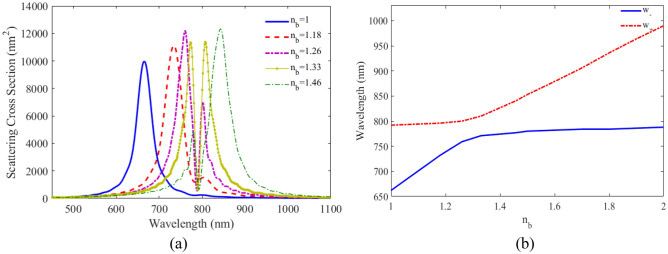
(**a**) SCS versus wavelength for plexciton rod nanoshell with different RI of the environment. (**b**) The variation of maximum wavelengths versus RI of plexciton rod nanoshells.

To investigate the performance of the proposed plexciton rod nanoshell as a nanosensor, the scattering spectra for different RIs of surrounding media (n_b_) is calculated and the result is plotted in Fig. 11a. The peak position of LSPR shifts to higher wavelengths as the RI of surrounding media increases. The variation of the wavelengths of the two split peaks of scattering spectra versus the RI of the surrounding media for biological materials, such as, blood, muscle, etc.: from 1 to 2 is calculated and the result shown in Fig. 11b. It can be observed that the wavelength of two peaks redshift without crossing the 792 nm, which is the absorption wavelength of QE. Nanorod nanoshells have a higher sensitivity than spherical and elliptical nanoshells. This is because the nanorod shape has a larger cross-sectional area, which allows for more light to be coupled into the plasmonic mode.

### Plasmon-exciton coupling in cone like nanoshells

The last geometry studied in this paper is a plexciton cone like nanoshell consisting of Ag core with a height and a radius of 60 nm and 28 nm, respectively coated with a 5 nm of QE. The wavelength of QE is 762.5 nm. The designed plexciton nanostructure with the simulation space is shown in Fig. 1d.

**Figure 12:**
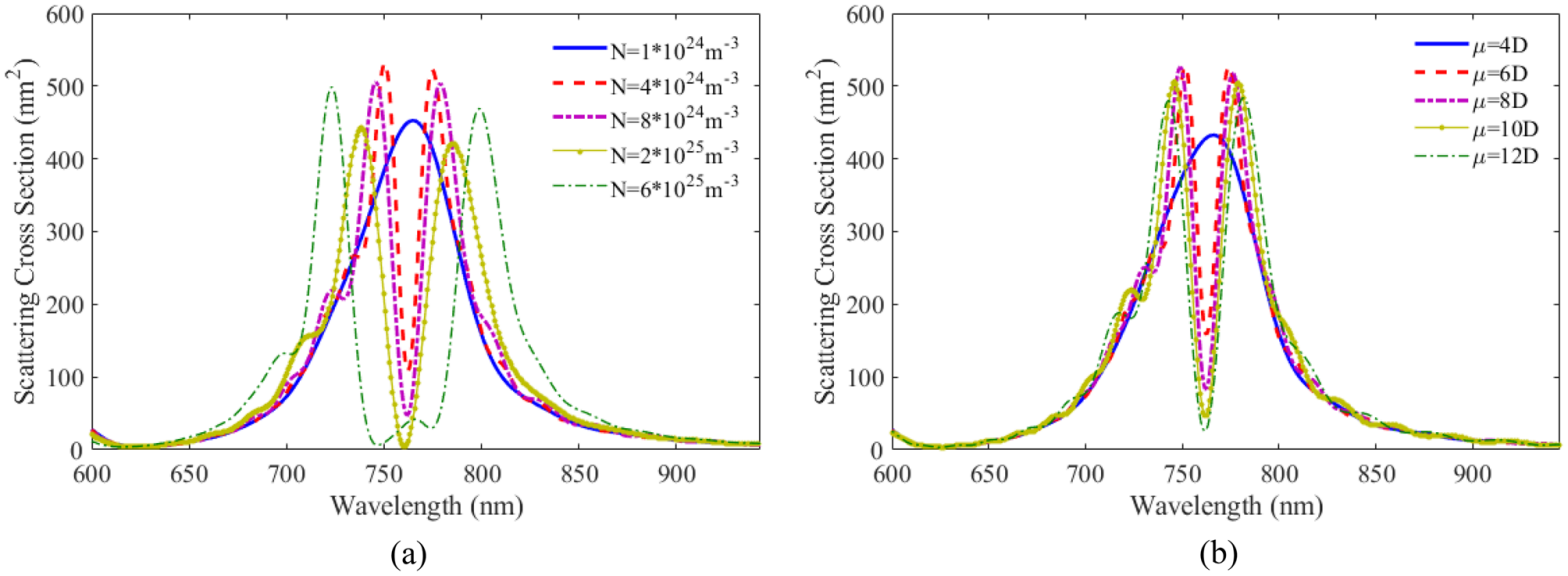
SCS versus wavelength for plexciton cone like nanoshell consisting of Ag core with a height and a radius of 60 nm and 28 nm, respectively coated with a 5 nm of QE with different (**a**) number densities, (**b**) dipole moments of QE.

Figure 12a shows the scattering spectra versus wavelength for plexciton cone like nanoshell with different number densities of QE. The distance between two peaks in the figure increases as the number density of the QE increases. A new peak is generated due to the enhance coupling of the QE and the nanostructure. In Fig. 12b the scattering spectra is plotted as a function of wavelength with different dipole moments of the QE. It can be seen that with an atomic dipole moment of less than 6D, there is no coupling between the QE and metallic nanoparticles. With increasing dipole moment, the coupling increases, and the scattering height reduces. Cone-like nanoshells have the highest sensitivity of all the nanoshell geometries. This is because the cone-like shape allows for even more efficient coupling of light into the plasmonic mode.

### Sensivity

**Figure 13:**
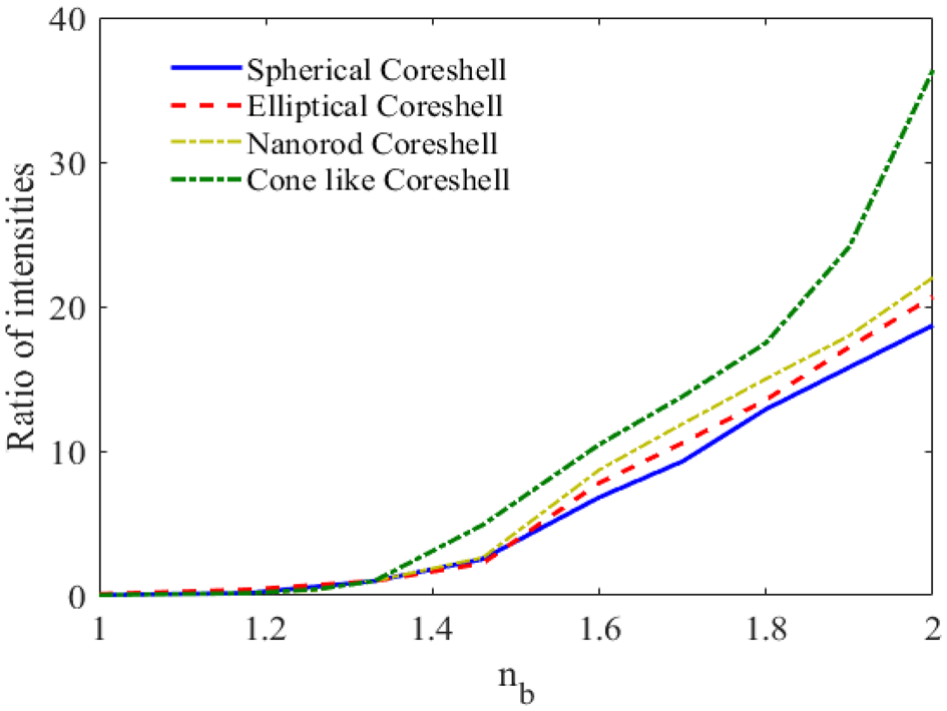
The ratio of two peaks in scattering curve versus RI for different nanoshell geometries.

Finally, in order to compare the designed nanosensors with different nanoshell geometries including spherical, elliptical, nanorod and cone like nanoshell, the ratio of the two peaks of the scatter spectrum is plotted versus the RI of the environment for different nanoshell geometries in Fig. 13. As can be seen, the cone like nanoshell with Ag core and QE shell has the highest sensitivity. Nanoshells with spherical, elliptical, and rod-shaped geometries also show similar sensitivities.

As can be seen, the cone-like nanoshell has the highest sensitivity. This is because the cone-like shape allows for more efficient coupling of light into the plasmonic mode, which leads to a stronger signal. The spherical and elliptical nanoshells have lower sensitivities because they do not couple light as efficiently into the plasmonic mode. However, in the case of spherical nanostructure, the surface morphology of spherical NPs, including their roundness and randomly faceted surfaces, can significantly affect their optical and plasmonic properties, as well as their interaction with QEs^74^. The nanorod nanoshell has a higher sensitivity than the spherical and elliptical nanoshells, but not as high as the cone-like nanoshell. This is because the nanorod nanoshell has a smaller cross-sectional area, which reduces the amount of light that can be coupled into the plasmonic mode. It is important to note that the sensitivity of a nanosensor is not the only factor that determines its performance. Other factors, such as the size and shape of the nanosensor, the type of analyte being detected, and the background noise level, also play a role. However, the geometry of the nanoshell is an important factor that can be used to improve the sensitivity of a nanosensor.

## Conclusions

In this paper, we propose and design plexciton nanoshells with various geometries for biosensing applications. We investigate the optical properties of the proposed nanoshells, including scattering and absorption spectra. We study how electromagnetic radiation interacts with atoms that have multiple energy levels, which contain both the spatial and temporal variation of the local electric field. We have used 3D-FDTD method to solve the coupled Maxwell-Liouville equations for different geometries. We investigated the effects of nanoshell geometries, sizes, and QE parameters on the sensitivity of nanosensors to changes in the RI of the environment for biological materials. We also study this effect of key parameters, including the geometrical parameters of the core and shell of the nanoshells, the RI of the surrounding media, and the QE parameters on the optical properties of the proposed nanostructure. By comparing the sensitivity of the designed nanoshells with various geometries, including spherical, elliptical, nanorod, and cone-like nanoshells, we find that the cone-like nanoshell has the highest sensitivity among all proposed plexciton nanosensors. The sensitivity of a nanosensor is determined by the geometry of the nanoshell, as well as other factors such as the size and shape of the nanosensor, the type of analyte being detected, and the background noise level. Under specific conditions, two sharp, deep LSPR peaks were evident in the scattering data. These characteristic features are respected as signatures in nanosensors that require rapid, non-invasive response.

## Data Availability

All data generated or analyzed during this study are included in this published article.
